# Biopsychosocial Markers of Body Image Concerns in Patients with Head and Neck Cancer: A Prospective Longitudinal Study

**DOI:** 10.3390/curroncol29070353

**Published:** 2022-06-22

**Authors:** Justine G. Albert, Christopher Lo, Zeev Rosberger, Saul Frenkiel, Michael Hier, Anthony Zeitouni, Karen Kost, Alex Mlynarek, Martin Black, Christina MacDonald, Keith Richardson, Marco Mascarella, Gregoire B. Morand, Gabrielle Chartier, Nader Sadeghi, Khalil Sultanem, George Shenouda, Fabio L. Cury, Melissa Henry

**Affiliations:** 1 Division of Experimental Medicine, Faculty of Medicine, McGill University, Montreal, QC H3A 0G4, Canada; justine.albert@mail.mcgill.ca; 2 Department of Psychology, College of Healthcare Sciences, James Cook University, Singapore 387380, Singapore; chris.lo@jcu.edu.au; 3 Department of Psychiatry, Temerty Faculty of Medicine, University of Toronto, Toronto, ON M5S 1A1, Canada; 4 Social and Behavioural Health Sciences, Dalla Lana School of Public Health, University of Toronto, Toronto, ON M5S 1A1, Canada; 5 Gerald Bronfman Department of Oncology, Faculty of Medicine, McGill University, Montreal, QC H3A 0G4, Canada; zeev.rosberger@mcgill.ca (Z.R.); fabio.cury@mcgill.ca (F.L.C.); 6 Department of Psychology, McGill University, Montreal, QC H3A 0G4, Canada; 7 Lady Davis Institute for Medical Research, Jewish General Hospital, Montreal, QC H3T 1E2, Canada; michael.hier@mcgill.ca; 8 Department of Otolaryngology—Head and Neck Surgery, Faculty of Medicine, McGill University, Montreal, QC H3A 0G4, Canada; saul.frenkiel@mcgill.ca (S.F.); anthony.zeitouni@mcgill.ca (A.Z.); karen.kost@mcgill.ca (K.K.); alex.mlynarek@mcgill.ca (A.M.); martin.black@mcgill.ca (M.B.); keith.richardson@mcgill.ca (K.R.); marco.mascarella@mcgill.ca (M.M.); gregoire.morand@mail.mcgill.ca (G.B.M.); nader.sadeghi@mcgill.ca (N.S.); khalil.sultanem@mcgill.ca (K.S.); george.shenouda@mcgill.ca (G.S.); 9 Department of Otolaryngology—Head and Neck Surgery, Jewish General Hospital, Montreal, QC H3T 1E2, Canada; 10 Department of Otolaryngology—Head and Neck Surgery, McGill University Health Centre, Montreal, QC H4A 3J1, Canada; 11 Department of Nursing, Jewish General Hospital, Montreal, QC H3T 1E2, Canada; christina.macdonald2@mail.mcgill.ca (C.M.); gchartier@jgh.mcgill.ca (G.C.); 12 Department of Radiation Oncology, Jewish General Hospital, Montreal, QC H3T 1E2, Canada; 13 Department of Radiation Oncology, McGill University Health Centre, Montreal, QC H4A 3J1, Canada; 14 Louise Granofsky Psychosocial Oncology Program, Jewish General Hospital, Montreal, QC H3T 1E2, Canada

**Keywords:** body image, cancer oncology, psycho-oncology, head and neck Cancer, longitudinal

## Abstract

(1) Background: Patients and survivors of head and neck cancer (HNC) are at a high risk of developing body image concerns. Despite the prevalence of body image concerns in patients with HNC, there is a lack of longitudinal research exploring the wide array of its associated determinants. The current longitudinal study examined the determinants and longitudinal course of body image dissatisfaction in patients with HNC. (2) Methods: Patients participated in Structured Clinical Interviews and self-administered questionnaires at four time-points: (T1) upon cancer diagnosis, (T2) at 3 months post-diagnosis, (T3) at 6 months post-diagnosis, and (T4) at 12 months post-diagnosis. They also underwent a disfigurement rating on an objective scale. (3) Results: Two hundred and twenty-four patients participated in our study. Fourteen percent to twenty-eight percent of patients reported at least moderate body image concerns across time points, with the lowest rates at baseline and the highest at 3 months (T1). It was found that patients more predisposed to developing higher levels of body image concerns presented physical markers (i.e., advanced cancer stage, lower physical functioning, higher disfigurement), psychosocial markers (i.e., higher depression, higher anxiety, and higher levels of coping with denial), and health disparities (i.e., younger age, female sex, French language, and marital status, with divorced and widowers most affected). (4) Conclusions: The findings of this study highlight the multifaceted nature of body image concerns in patients with HNC and its biopsychosocial determinants. Clinicians should pay specific attention to these biopsychosocial markers in their clinics to predict high levels of body image concerns and tailor communication/refer for support accordingly.

## 1. Introduction

Cancer and its associated treatments often cause patients to undergo physical, functional, and psychological changes that affect their appearance and identity [1]. Some examples of changes include the loss of hair and body parts, scarring, weight changes, disfigurement, and functional disabilities [2]. These modifications can alter a patient’s perception of self and cause body image concerns [1]. 

Although many definitions of body image exist, the definition most commonly used in cancer-related body image research and employed in this study is body image as an individual’s subjective thoughts and perceptions of their physical appearance [3]. The concept of body image is most commonly researched in adolescent and young adult populations with eating disorders and is less clearly outlined in the context of cancer [4,5]. Body image concerns in adults with cancer have distinct characteristics that differ from individuals with eating disorders, leading to different treatments [4,5]. The main characteristics of body image concerns in adults treated for cancer are: (1) perceiving a change in appearance and feeling displeasure with the perceived change, (2) experiencing a decline in functional abilities, and (3) experiencing psychological distress about the perceived changes in appearance and/or function [6].

Considering the broad definitions of body image concerns in oncology, patients with different cancer types and stages are susceptible to developing body image concerns, including breast, gynecological, testicular, colorectal, and head and neck cancers. Body image concerns are amplified in young adolescent and young adult patients [4]. In patients with breast cancer, these concerns have been linked to chronic fatigue, low sexual functioning, poor physical and mental health, increased levels of depression, social anxiety, low self-esteem, and overall reduced quality of life [5,7]. 

Patients and survivors of head and neck cancer (HNC) are also highly susceptible to body image concerns due to the visible physical and functional disturbances these types of cancer often cause [1,2]. Previous research suggests that 89% of patients with HNC present with some level of body image concerns immediately post-treatment (3 months), with 20% mild to moderate and 9% moderate to severe levels, respectively [6]. HNC are malignant tumours arising in the lining surfaces of the oral cavity, salivary glands, pharynx, larynx, sinuses, and nasal cavity, which are often diagnosed in advanced stages and require invasive treatments that lead to altered facial appearance, pain, and the changes on basic functional abilities, such as swallowing, speaking, and breathing [1,2]. The treatment recovery for patients with HNC can take from 12 to 36 months on average and it can include long-term sequelae [8]. 

The high prevalence of body image concerns in patients with HNC contrasts with the lack of research on the topic in this population, with most studies focusing on patients with breast cancer [5,7]. Furthermore, past studies of body image concerns in patients with HNC have mainly been cross-sectional, precluding the identification of early determinants of body image concerns in survivors [4,5,7]. Longitudinal studies are needed to better predict upon HNC diagnosis who will develop body image concerns into survivorship to better support patients. This is especially important as the social discomfort caused by body image concerns can lead to social isolation, which causes greater rates of morbidity and mortality. Having a good perceived body image promotes better treatment and disease outcomes and a better ability to cope with the cancer diagnosis [1,2]. 

**Figure 1:**
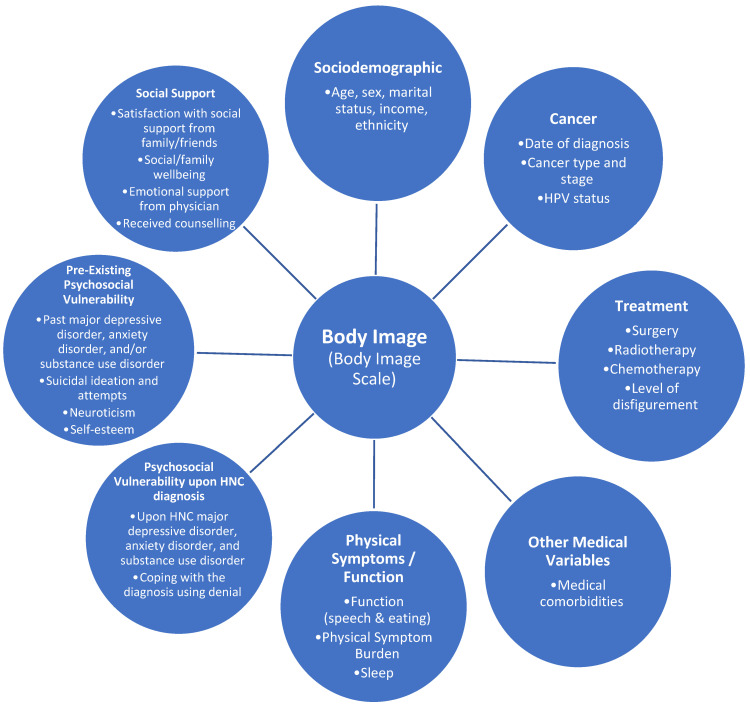
Conceptual model of predictors of body image concerns in patients with HNC.

The current longitudinal study examined the determinants and longitudinal course of body image dissatisfaction in head and neck cancer patients from baseline to 1 year. Our conceptual model was based on the Wilson and Cleary model [9] combined with the Diathesis–Stress model [10,11]. The Wilson and Cleary model includes both psychological and biological determinants of health-related quality of life [9]. The Diathesis–Stress Model suggests that a person’s mental health results from both their individual vulnerability and their environmental stress [10,11]. Our conceptual model includes seven main components: sociodemographic, cancer- and treatment-related variables, other medical variables, physical symptoms/function, pre-existing and upon cancer psychological vulnerability, and social support (see Figure 1).

## 2. Materials and Methods

### 2.1. Participants 

Patients eligible for the study had to be 18 years and older, be cognitively capable of providing informed consent according to physicians and had to have a Karnofsky Performance Scale (KPS) score of at least 60, and/or survival of at least six months, based on the medical team. Participants were all recruited before the onset of treatment, within two weeks of being diagnosed with the first occurrence of primary HNC. All patients were staged according the AJCC TNM staging system, 8th edition.

Eligible patients were identified by physicians or nurses of the Department of Otolaryngology-Head and Neck Surgery at McGill University-affiliated hospitals (Jewish General Hospital, McGill University Health Centre—Montreal General Hospital, and Royal Victoria Hospital sites). A log was kept of study participants upon enrolment to track attrition over time. 

### 2.2. Procedure 

The current study received full ethics approval from the McGill University Faculty of Medicine’s Institutional Review Board, #A05-B24-10B. Patients participated in Structured Clinical Interviews for DSM-IV Diagnoses (SCID-I) [12] and completed self-administered questionnaires at 4 different time points: (1) upon cancer diagnosis, (2) at 3 months post-diagnosis, (3) at 6 months post-diagnosis, and (4) at 12 months post-diagnosis. We used written means of communicating with patients when needed, in consideration of their speech function/impairments. Observer-rated disfigurement was also measured at baseline (i.e., upon cancer diagnosis/pre-treatment) and 3 months post diagnosis, with the 3 month timepoint carried forward into 6 and 12 months and after HNC treatments. The SCID-I interviews were conducted by the research coordinator, who was trained and supervised by the principal investigator.

### 2.3. Measures 

The questionnaires took approximately 60 minutes to complete at baseline and 45 minutes at 3 months, 6 months, and 12 months. Disfigurement rating was only assessed at baseline and 3 months, with the 3-month timepoint carried forward into 6 and 12 months. The other measurements were assessed at baseline, 3 months, as well as 6 and 12 months. 

Primary Outcome. The Body Image Scale (BIS) is a 10-item measure that examines the way patients perceive their bodily appearance. The BIS is the most common measure used for body image research, specifically in patients with cancer. The questions of the BIS include 4-point Likert type scales where participants indicate their level of body image concerns as 0 = not at all, 1 = a little, 2 = quite a bit, and 3 = very much [13]. The internal consistency of the BIS is 0.93, the test–retest reliability is 0.70, and a cut-off score of ≥10 on the BIS indicates clinically significant levels [14,15].

Determinants. Self-administered questionnaires were used to collect sociodemographic data, including age, sex, work status, education, marital status, living situation, and individual and family income. Medical variables were assessed through medical chart review and included data about cancer type, tumour site, cancer stage, medical comorbidities, HPV status, performance status (ECOG Status), and treatments received (i.e., surgery, radiation therapy, chemotherapy) [16]. We used radial forearm free-flap treatment as a marker of surgery extent. Counseling/therapy participation and psychiatric medications were also assessed through a chart review and questions in the questionnaire. 

The Observer-Rated Disfigurement Scale for Head and Neck Cancer was used to evaluate levels of disfigurement at baseline and at the 3-month follow-up [17].

The Functional Assessment of Cancer Therapy—General (FACT-G) [18] measured quality of life. Physical symptoms were measured by the FACT-G Physical Wellbeing subscale (a 7-item scale with a score ranging from 0–28, internal consistency: >0.70) and the FACT-Head and Neck Module (FACT-HN). Quality of sleep was measured using item F5 of the FACT-G. Patients complete each question on the FACT-G or FACT-HN using a 5-point Likert type scale with scores from 0 to 4, where higher scores signify better quality of life [19]. Functional status was measured using the Eastern Cooperative Oncology Group (ECOG) scale [20] and FACT-HN items #7, #11 (swallowing), and #10 (speaking) [19]. 

The Structured Clinical Interview for DSM-IV diagnoses were used to assess pre-existing psychosocial vulnerabilities, such as anxiety disorder, depressive disorder, or substance use disorder before and upon cancer diagnosis (the inter-rater reliability for symptoms is 0.75 and on diagnoses is 0.90) [12]. Self-esteem was measured using the 10-item Rosenberg Self-Esteem Scale, with the total score ranging from 0 to 40 and higher scores representing higher levels of self-esteem (test–retest reliability is 0.85–0.88; internal consistency is 0.92) [21]. 

Pre-treatment levels of anxiety and depression were measured using the 14-item Hospital Anxiety and Depression Scale (HADS), with the total score ranging from 0 to 42 and higher scores indicating greater levels of anxiety and depression (test–retest reliability is >0.80; internal consistency is 0.78–0.93) [22]. Past and current suicidal ideation were measured using the 21-item Beck Scale for Suicidal Ideation, with the total score ranging from 0 to 42 and higher scores indicating higher levels of suicidal ideation (internal reliability is 0.94) [23]. Alcohol misuse was measured using the 5-item Rapid Alcohol Problems Screen, with higher scores indicating a higher level of alcohol misuse [24]. Drug misuse was measured using the 10-item Drug Abuse Screening Test, with the total score ranging from 0 to 10 and higher scores indicating a higher level of drug misuse (internal consistency reliability is 0.92) [25].

Neuroticism was measured using the 12-item Eysenck Personality Inventory—Neuroticism Subscale, with the total score ranging from 0 to 36 and higher levels indicating greater levels of neuroticism (internal consistency is 0.80–0.84) [26]. Use of denial and avoidance to cope with the cancer diagnosis was measured using the 2-item Brief COPE denial subscale with scores ranging from 2 to 8 and higher scores indicating higher denial-based coping (internal consistency is 0.54) [27].

Social Support was measured using the 12-item Social Support Questionnaire with a total score ranging from 0 to 90 with higher scores indicating greater levels of social support (test-retest reliability is 0.90; internal consistency is 0.90–0.93) [28]. Childhood abuse was measured using the Statistics Canada Canadian Incidence Study of Reported Child Abuse and Neglect (CIS) (i.e., physical, psychological, sexual abuse, and neglect) [29]. The supportive care needs of participants were measured using the 34-item Supportive Care Needs Survey—Short Form SCNS-SF34, with the total score ranging from 34 to 170 and higher scores representing greater levels of unmet care needs (internal consistency is 0.86–0.96) [30].

### 2.4. Statistical Analyses 

Descriptive statistics were calculated to characterize the sample. The main analysis was a linear mixed model fitting 2-level growth curves describing body image across the 4 assessments taken at baseline (T0), 3 months (T1), 6 months (T2), and 12 months (T3). First, an unconditional model was fit to assess the intraclass correlation (ICC), indicating the percent variation attributable to individual differences (i.e., between subjects). A second model with time as the main predictor was used to fit a growth curve of the pattern of change over time. We tested for linear, quadratic, and cubic trends and for a random effect of time to assess individual differences in the slope or degree of change. A third explanatory model was built, in which other time-dependent and time-invariant factors were tested as proximal determinants of body image.

## 3. Results

Two hundred and twenty-four patients participated in our study (71.5% participation) between September 2012 and September 2015. Reasons for declining participation included having no time (38%), having other priorities (20%), or participation would be felt as too demanding emotionally (18%) or time-wise (13%). Our sample had a mean age of 63, with a third of the sample being female and 73% of participants had a late stage of HNC. Most predominant tumour sites were oropharyngeal cancer (37%), followed by oral cancer (21%), and laryngeal cancer (17%). Forty-two percent of cancers were HPV-positive. Most participants were married, and half of the participants identified as French speakers (see Table 1). Ninety-seven percent of patients had finished treatments at 3 months follow-up. Based on the Body Image Score cut-off greater or equal to 10, the percentage of individuals reporting at least moderate body image dissatisfaction ranged from 14% to 28% across time points, with the lowest being at baseline and the highest at 3 months (T1) (see Table 1 for sociodemographic, medical, and clinical characteristics).

Participant attrition at 3 months, 6 months, and 12 months was not found to be due to sociodemographic (sex, age) and medical (stage) differences at baseline; and attrition at 3 months was not due to psychological differences. Patients having dropped out at 6 and 12 months were found to have higher levels of psychological distress at baseline (*p* = 0.09 and *p* = 0.048, respectively; depression *p* = 0.006–0.009; anxiety *p* = 0.31–0.47). Those who did not complete follow-up at 3 months presented with significantly lower ECOG functioning at baseline (*p* < 0.005; 6 and 12 months *p* = 0.41–0.72). There was a trend for 3-month disfigurement rates to be higher in the drop-out group at 12 months (*p* = 0.09) but not at 6 months (*p* = 0.62–1.0). Based on the unconditional model variances, the interclass correlation (ICC) was calculated to be 0.54, meaning that 54% of the variation between time points was attributable to between-subjects (intercepts) versus within-subjects (residual) (see Table 2 for results across models). After fitting a predictive model focusing on temporal changes only, we found significant linear (Time) and curvilinear (quadratic and cubic) effects (see Table 2). 

**Figure 2:**
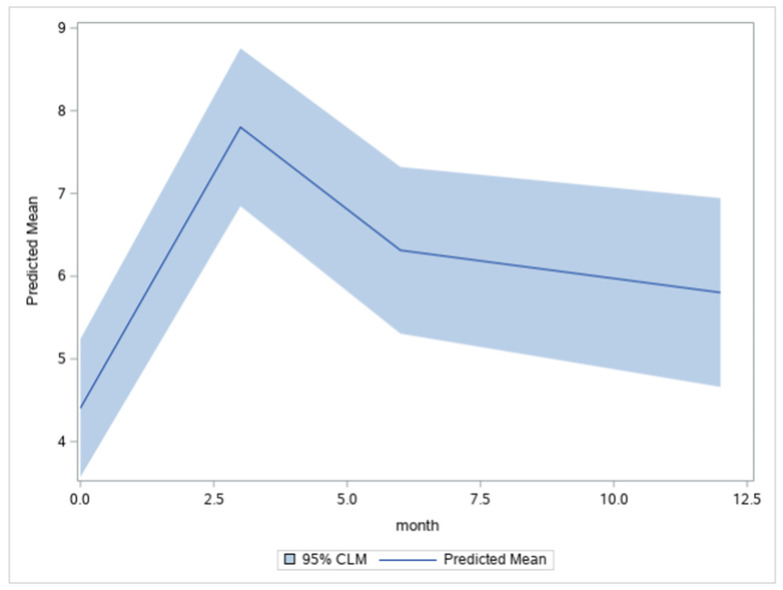
Expected growth curve plotted as a function of months. Note. The predicted means (and 95% Confidence Limits for the Mean) were: T0 = 4.41 (3.57, 5.24), T1 = 7.80 (6.85, 8.76), T2 = 6.31 (5.31, 7.32), and T3 = 5.80 (4.66, 6.94).

Demonstrated in Figure 2, which is a plot of the expected growth curve or the averaged individual trajectory for the sample, we found that the profile was characterized by a substantial increase in BIS from T0 to T1 (d = 0.59), followed by a reduction from T1 to T2 (d = 0.26), with stability at T3 (d = 0.09). Meaning that individuals are expected to have the most significant body image concerns at 3 months and that body image concerns halve in severity at 6 months and stabilize at 1 year but do not recover to baseline levels of body image concern. 

**Figure 3:**
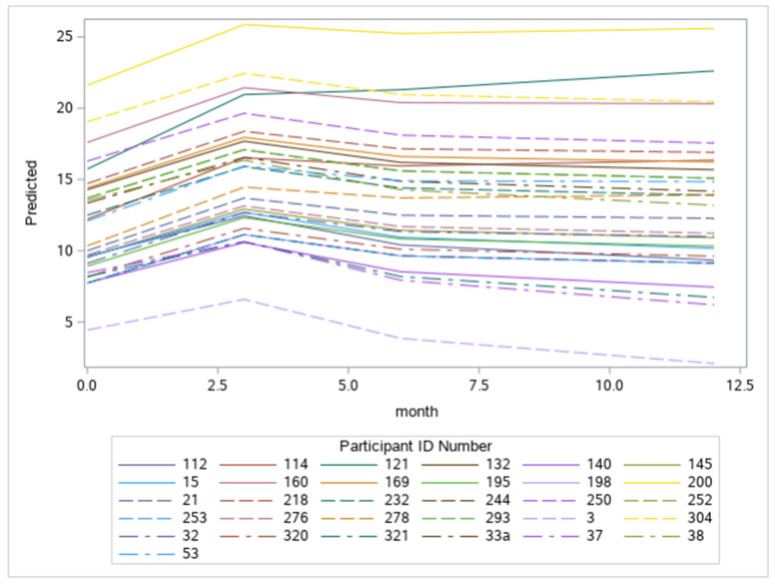
Examples of predicted individual growth curves as a function of months.

There was also a significant random effect of time, indicating variation in the linear slopes across individuals. Figure 3 exemplifies the predicted individual growth curves to give a sense of this variation. Note that the growth curves differ in steepness, and individuals may enter at different baseline levels. 

Based on the explanatory model of the determinants of body image over time created for this study (see Table 2), there were 14 significant effects. Firstly, body image dissatisfaction over time was associated with poorer physical functioning, both overall and specific to the head and neck region, and with disfigurement. 

**Figure 4:**
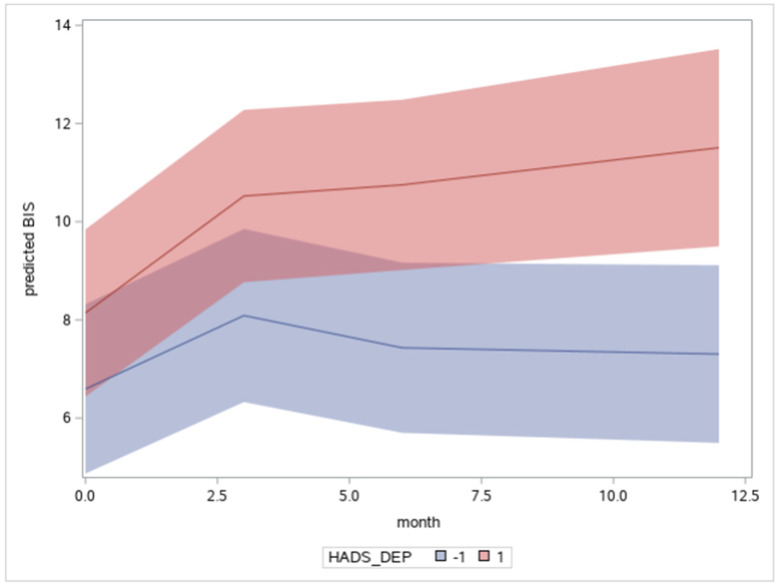
Plot of the Time × Depression interaction as a function of months. Note. The blue line estimates the growth curve of individuals with depression scores −1 SD below the mean and the red line +1 SD above the mean, controlling for other factors. Shaded regions indicate the 95% confidence limits.

Body image concerns were also related to levels of depression and anxiety. We found a significant Time x Depression interaction, indicating that body image dissatisfaction continued to worsen for individuals with persistent depressive symptoms over time (see Figure 4). In Model 3, the random effect of Time was reduced to non-significance (see Table 2), mainly by the inclusion of the Time x Depression interaction. This would suggest that individual differences in the Time slope are related to unresolved depressive symptoms, which exacerbate the severity of body image disturbance, preventing the adaptive reduction in body image concerns that might otherwise be expected to occur. 

Furthermore, individual differences in body image disturbance were associated with younger age, female gender, more advanced cancer staging, denial, marital status, and being a French language speaker. Concerning marital status, additional contrasts clarified that single individuals were least affected by body image concerns than married, divorced, and widowed persons (*p* = 0.008), and married persons were less affected than divorcees and widowers (*p* = 0.02). However, divorcees and widowers did not differ from each other. 

## 4. Discussion

Using the HNC specific conceptual model, the current longitudinal study examined the determinants and longitudinal course of body image concerns in patients with head and neck cancer from baseline to 1 year. The study aimed to investigate whether an individual’s body image concerns changed over time and if there is a specific time point where people are more susceptible to experiencing high levels of body image concerns. The study also assessed whether the variation in participants was attributable to individual differences.

The current study presented a model of normative trajectory for body image concerns in HNC over 1 year. We found that the most significant disturbance, of medium effect size, occurs at 3 months. Although there is a subsequent adaptation to the effects of HNC and its treatments, body image concerns do not recover to baseline levels over time. These findings align with past research underlining body image concerns as initially worsening post-treatment, followed by slight improvement over the months following treatment, with many patients never returning to pre-treatment levels [13,31]. It has also been found that body image concerns often either improve or stabilize over time as patients undergo further surgical reconstruction 6 to 12 months after their initial surgery to optimize their appearance and functional abilities [13,31]. Alternatively, the opposite has also been found, with levels of body image concerns worsening over time as patients focus less on the fear of cancer recurrence and more on their appearance and the other long-term visible consequences of their cancer [31,32].

Our study results indicate that half of the variation in body image scores is attributable to individual differences and half to intra-individual changes over time. Notably, personological factors loom large in predicting adaptation to HNC, but it remains unclear in the current scientific literature which personological factors specifically affect body image. Allen and Walter [20] found high levels of neuroticism and low levels of extraversion to be most associated with an individual experiencing body image concern. Moreover, in our study, there was some variation in the slope of individual changes over time, which are related to the experience of persistent depressive symptoms. Past research has found that patients who experience pre-treatment depressive symptoms often report higher levels of body image concerns, which could be associated with this current study finding [1,2].

Previous studies have focused on psychosocial factors, such as depression and anxiety, that render certain patients susceptible to body image concerns, mainly using conceptual models that include the moderating roles of coping strategies, patient surgical expectations, and body image investment [13]. Using our HNC-specific model conceptualizing body image as a function of personal vulnerability and contextual stress, it was found that changes in levels of body image concerns may be explained by a host of biopsychosocial factors, including physical markers (i.e., disfigurement, changes in physical functioning, and cancer staging), psychosocial markers (i.e., depression and anxiety, individual differences in coping with denial and avoidance), and social disparities (i.e., age, gender, language, and marital status).

Physical markers are important drivers of body image concerns. Disfigurement as a determinant of body image concerns is in line with previous studies having found an association of higher shame and lower appearance satisfaction with lower body image [5,7]. Radical surgery was found in previous studies to change one’s appearance and have an impact on body image concerns [13]. Physical functioning as a determinant is also in accordance with past research, which found impairments in speech and eating as predictors of body image concerns [6,31,32]. Having a positive body image often means placing less value on physical appearance and more on physical ability and functionality [6,20,33]. Patients who are less physically able because of pain or other symptoms can experience an altered perception of their bodies [6,20,33]. Functional impairment can cause an individual to feel uncomfortable and self-conscious due to their physical limitations and a feeling that something is wrong with their body [6,20,33]. Cancer staging also plays a part in driving body image, with patients with advanced-stage cancer having also previously been found to be often more dissatisfied with their body image compared to those with early-stage cancer [5,34]. Future research could help better elucidate the specific elements of disfigurement, physical functioning, and cancer staging that most strongly determine levels of body image concerns, as this would aid in developing targeted interventions. One could conceptualize issues around body image concerns using a larger framework for function, encompassing bodily functions and structures, limitations in activities of daily living, and participation in life roles [35]. It may well be that the changes one experiences in one’s body within the context of a health condition feeds into the existential concerns brought about by advanced cancer. One would also want to study the interface between visible bodily changes and interpersonal relationships, including health-related stigma, examining individual components of stigma, such as anticipated stigma (perceived), internalized stigma (self-stigma), and experienced stigma (discrimination), as well as the institutional sources of stigma (enacted stigma/discrimination and negative attitudes/prejudices).

Psychosocial markers are important drivers of body image concerns. Depression and anxiety were found to be determinants of body image concerns, in accordance with past research [1,2]. Depressogenic cognitive processing styles are filters through which people perceive self, others, and the future in negative ways. These patterns of thinking are related to increased vulnerability to developing depression in the face of life stress, which, in turn, can complicate people’s readjustment to bodily changes and merits further intervention to facilitate body reintegration post-treatment [36]. As for denial, it is defined in patients with cancer as the unconscious defense against painful aspects of reality and is often associated with distress and poorer psychological functioning [37]. Coping with denial and avoidance in the face of cancer can affect information processing and contribute to unrealistic expectations as to short- and longer-term treatment impacts. It is also possible that patients typically using avoidance are especially anxious as well as perhaps unprepared to cope with the reality of the bodily changes they face, as this would require approach-based coping. The link between coping with denial, adjustment to changes in the body, acute stress, and ability for self-care is so important in the advent of major surgeries that it would merit further research.

Social disparities are important drivers of body image concerns. Individual differences of body image concerns were associated with younger age, female gender, marital status, and being a French language speaker. While past research on this topic is limited, age has been found to be a moderator for psychosocial distress related to disfigurement [5]. Patients younger than 65-years of age with HNC are more concerned about their appearance and, in turn, suffer body image concerns more often than older patients [38]. Sex has also been associated with the level of body image concern in past research, as women, specifically younger women, often experience more body image concerns and reduced overall well-being due to these concerns [38]. Being a French language speaker was a determinant of experiencing body image concerns. This may merit further attention as patients with HNC need to travel sometimes long distances to the hospital from rural areas to be treated in McGill University-affiliated oncology centers, in which English is the primary language. Finally, the current study found marital status to be associated with body image, specifically that single individuals were the least affected by body image concerns compared to married, divorced, and widowed persons, where married patients were less affected by body image disturbance than divorcees and widowers. There is very little past research looking at the connection between romantic relationships and body image in the context of cancer. However, Paterson and colleagues [5] found that greater emotional support from one’s partner is often associated with fewer sexual difficulties after treatment among breast cancer survivors and that patients who perceived themselves to be in a positive relationship with a partner experienced better body image. It has also been found that in patients identifying as female and relationship satisfaction predicted their perceptions of their partners acceptance of their appearance, potentially affecting their own body image [24]. This may be related to the fact that impaired sexual functioning or a lack of intimacy overall are associated with body image concerns and often time sexual difficulties are correlated with one’s marital or relationship status [5]. However, the fact that single patients were the least affected by body image concerns has not been discussed in past research to our knowledge and may be an interesting topic to further research.

The findings of the study demonstrate the multifaceted nature of how body image is experienced in patients with HNC and the necessity of using a Diathesis–Stress model that touches on biopsychosocial factors. The determinants found to be associated with body image concerns in the current study related to each component of our HNC-specific conceptual model. Note that when constructing the model, we tested many disease-related and psychosocial factors, including tumor site, HPV status, work status, education, and living situation to see if they would remain in the model but the model described here was the best that could be found. The other variables were non-significant. However, they may still exert influence on body image concerns, but through more complex pathways as mediated through some of the relatively proximal factors discussed here and would require a structural equation modeling approach to clarify. Lastly, the findings also highlight the time-frames where body image interventions are most needed.

### 4.1. Limitations 

While the current study added to body image research in the HNC population and is based on the first large cohort of patients with HNC, there remain limitations. For instance, this paper focused on characterizing the normative individual trajectory of body image concerns; however, the random effect of time was small on levels of body image concerns. Other studies may, therefore, seek to identify and classify latent subgroup trajectories. Furthermore, the analysis of the current study focused on building a model of proximal explanatory factors for body image issues, while future research in the field may seek to also examine longer chains of causal influence from distal to proximal factors. The Body Image Scale used in the study may also be a limitation, as although it was the preferred measure at the time of the study, the measure was created for breast cancer patients and may be missing items specific to the experience of patients with HNC. Future studies may choose to use the recently created and validated body image concern measure specific to HNC known as the FACT/McGill Body Image Scale—Head and Neck (FACT-MBIS) [39]. Finally, patients who dropped out presented certain characteristics (lower functional status) that may limit the study’s generalizability.

### 4.2. Clinical Implications

Based on our findings, clinicians should aim to develop more cancer-specific biopsychosocial markers to identify patients more susceptible to body image concerns. These biomarkers could be in the form of cancer-specific screening tools to assess disfigurement [17] or functional status [40,41], or a general screening tool such as the Edmonton Symptom Assessment System, followed by further patient-reported outcomes targeting anxiety and depression [42,43]. Physicians, nurses, and the allied health care team could use single-item scales in their practice as tools to pick up patients with higher levels of anxiety and depression [43,44,45] and tailor their approach to consent and prescribe psycho-oncology as part of pre-habilitation [46,47] and recovery-oriented protocols [48,49]. Clinicians should also be aware of the other individual risk factors for body image concerns, such as younger age, female gender, more advanced cancer stage, denial, marital status, and being a French language speaker. By identifying patients with HNC at high risk of developing body image concerns, clinicians can provide preventative and therapeutic interventions to patients who are most in need before high levels of cancer-related body image concerns develop [13,38]. Interventions could be integrated into the clinic, such as collaborative care approaches for distress [50] and cognitive behavioral therapy for appearance anxiety [51].

Furthermore, the findings regarding the individual trajectory of body image concerns can help inform the effective timing of intervention delivery. Based on our results, body image concerns are the highest at 3 months, which is when patients will need the most support and therapy. By providing more targeted and timely interventions, clinicians have the potential to reduce patient psychosocial distress, improve cancer treatment outcomes, and increase the patient’s overall quality of life [38]. One must not forget the importance of preparing patients well for a life after treatments through the use of communication tools that will allow patients to have realistic expectations vis-à-vis their surgery. Such approaches have already been developed, such as the teach-back method, proven to be effective to prepare patients for other medical procedures, including in general oncology [52,53].

## 5. Conclusions

Patients and survivors with head and neck cancer are at a high risk of developing body image concerns due to the visible physical and functional changes that often occur as a consequence of cancer related treatments. Based on the results of the current study, clinicians should provide the majority of supportive resources and body image interventions at the 3 month post-treatment mark, as this is when patients experience the highest levels of body image concerns. Clinicians may also want to specifically screen for bio-markers (i.e., advanced cancer stage, lower physical functioning, and higher levels of disfigurement), psychosocial markers (i.e., depression, anxiety, and coping with denial), and health disparities (i.e., younger age, gender, language, and marital status). Our findings will allow researchers and clinicians to build more targeted interventions and provide these resources in a timelier manner.

## Figures and Tables

**Table 1:** Sample characteristics.

Time-Dependent Variables	N Total Sample	Mean	SD
BIS T0	218	4.46	5.75
BIS T1	148	7.54	7.34
BIS T2	141	5.83	6.22
BIS T3	117	5.57	6.84
HADS ANXIETY T0	219	6.02	4.65
HADS ANXIETY T1	146	4.67	3.97
HADS ANXIETY T2	140	4.11	3.71
HADS ANXIETY T3	119	3.60	3.43
HADS DEPRESSION T0	219	3.72	3.99
HADS DEPRESSION T1	146	4.15	3.80
HADS DEPRESSION T2	141	3.46	4.42
HADS DEPRESSION T3	119	2.54	3.23
FACT PHYSICAL T0	222	23.15	5.52
FACT PHYSICAL T1	151	19.80	6.40
FACT PHYSICAL T2	144	23.17	4.67
FACT PHYSICAL T3	123	23.71	5.08
FACT HEAD NECK T0	217	35.89	7.80
FACT HEAD NECK T1	149	29.15	7.64
FACT HEAD NECK T2	143	33.88	7.03
FACT HEAD NECK T3	118	35.64	7.94
DISFIGUREMENT T0	222	1.40	1.18
DISFIGUREMENT T1	181	2.04	1.70
DISFIGUREMENT T2	126	1.85	1.28
DISFIGUREMENT T3	103	1.82	1.32
	**N Total Sample**	**Frequency**	**Percent**
Treatment—3 months	215		
-Surgery alone		37	17
-Radiotherapy alone		21	10
-Chemotherapy alone		5	2
-Surgery and radiotherapy		23	11
-Radiation and chemotherapy		99	46
-Surgery and radiotherapy and chemotherapy		30	14
Treatment—6 months	6		
-Surgery alone		3	50
-Radiotherapy alone		1	17
-Chemotherapy alone		1	17
-Surgery and radiotherapy		0	0
-Radiation and chemotherapy		1	17
-Surgery and radiotherapy and chemotherapy		0	0
Treatment—12 months	11		
-Surgery alone		5	39
-Radiotherapy alone		2	15
-Chemotherapy alone		2	15
-Surgery and radiotherapy		0	0
-Radiation and chemotherapy		2	15
-Surgery and radiotherapy and chemotherapy		2	15
Free flap	208	30	14
BIS T0 ≥ 10	218	31	14
BIS T1 ≥ 10	148	42	28
BIS T2 ≥ 10	141	28	20
BIS T3 ≥ 10	117	25	21
**Time-Invariant Variables**	**N**	**Mean**	**SD**
Age	224	62.94	11.70
COPE DENIAL	219	3.07	1.65
	**N**	**Frequency**	**Percent**
Female	224	72	32
French Language	224	115	51
Marital Status	224		
Married		142	63
Divorced		47	21
Single		23	10
Widowed		12	5
Stage III/IV	222	161	73
Tumour site	223		
Oropharynx		82	37
Oral		46	21
Larynx		37	17
Skin		15	7
Nasopharynx		11	5
Unknown Primary		12	5
Other		20	9
Cancer type—HPV+	223	94	42
Physical function—ECOG 2+	221	25	10

**Table 2:** Standardized Estimates (and Standard Errors) from 2-Level Growth Models Examining Changes in Body Image in Patients with Head and Neck Cancer.

	Model 1 (Unconditional)	Model 2 (Time Only)	Model 3 (Explanatory)
**Fixed Effects**			
*Level 1: Time-Dependent Factors*			
Time (T0, T1, T2, T3)	-	1.19 *** (0.19)	0.60 ** (0.21)
Time *Time	-	−0.81 *** (0.18)	−0.37 * (0.19)
Time *Time *Time	-	0.15 ** (0.039)	0.068 (0.041)
FACT PHYSICAL	-	-	−0.12 ** (0.044)
FACT HEAD NECK	-	-	−0.15 ** (0.043)
HADS ANXIETY	-	-	0.13 ** (0.046)
HADS DEPRESSION	-	-	0.12 * (0.056)
Time*HADS DEPRESSION	-	-	0.067 * (0.028)
DISFIGUREMENT	-	-	0.079 * (0.037)
*Level 2: Time-Invariant Factors*			
Age at Baseline	-	-	−0.13 ** (0.049)
Female	-	-	0.22 * (0.095)
Stage III/IV	-	-	0.24 * (0.10)
COPE DENIAL	-	-	0.12 ** (0.044)
Married ^†^	-	-	−0.36 (0.21)
Divorced ^†^	-	-	−0.14 (0.23)
Single ^†^	-	-	−0.59 * (0.25)
French Language	-	-	0.19 * (0.086)
**Error Variance**			
Intercept	0.551 *** (0.072)	0.547 *** (0.072)	0.203 *** (0.037)
Time	-	0.025 * (0.012)	0.014 (0.009)
Residual	0.463 *** (0.033)	0.371 *** (0.030)	0.314 *** (0.026)
**Model Fit**			
AIC	1611.4	1572.3	1181.3
BIC	1618.2	1582.6	1251.9

^†^ Reference category was Widowed. * *p* ≤ 0.05; ** *p* ≤ 0.01; *** *p* < 0.0001.

## Data Availability

The data presented in this study are available by request to the authors.
